# When genomic medicine reveals misattributed genetic relationships—the debate about disclosure revisited

**DOI:** 10.1038/s41436-018-0023-7

**Published:** 2018-06-14

**Authors:** C. F. Wright, M. Parker, A. M. Lucassen

**Affiliations:** 1 Institute of Biomedical and Clinical Science, University of Exeter Medical School, Royal Devon and Exeter Hospital, Exeter, UK; 2 The Wellcome Centre for Ethics and Humanities/Ethox Centre, Nuffield Department of Population Health, University of Oxford, Oxford, UK; 3 Clinical Ethics and Law, Faculty of Medicine, University of Southampton, Southampton, UK

**Keywords:** disclosure, incidental finding, non-paternity, family trio, genome sequencing

## Abstract

**Purpose:**

Accidental discovery of misattributed parentage is an age-old problem in clinical medicine, but the ability to detect it routinely has increased recently as a result of high-throughput DNA sequencing technologies coupled with family sequencing studies. Problems arise at the clinical–research boundary, where policies and consent forms guaranteeing nondisclosure may conflict with standard clinical care.

**Methods:**

To examine the challenges of managing misattributed parentage within hybrid translational research studies, we used a case study of a developmentally delayed child with a candidate variant found through a large-scale trio genome sequencing study in which data from unrelated samples were routinely excluded.

**Results:**

We discuss whether genetic parentage should be explicitly confirmed during clinical validation, thus giving greater weight to the diagnosis according to American College of Medical Genetics and Genomics variant interpretation guidelines, and what tensions this approach would create.

**Conclusion:**

We recommend that the possibility of finding and disclosing misattributed parentage should be addressed during the consent or pretest counseling process, and that clinical relevance should determine whether or not to disclose results in the clinic. This proposition has implications for research governance, and implies that it may not always be possible to uphold nondisclosure commitments as investigations move from research to clinical care.

## INTRODUCTION

Rapid developments in high-throughput DNA sequencing technologies, resulting in increased speed and decreased cost of analysis, have been well documented. Many hundreds of new disease-causing or predisposing genes have been identified in the past decade, leading to a marked increase in the number of diseases for which the molecular etiology is known.^1,2^ Many diseases have heritable subtypes, making these developments relevant to all medical specialities.^3^ Increasingly, genetic approaches to diagnosis use a single assay, in which either all the genes in the genome (the exome) or the entire genome is sequenced at once. To improve the interpretation of genomic variation, a comparison with the genetic code in close relatives can help to distinguish pathogenic from unknown or background variation. One commonly used method in pediatrics is trio testing, where a child’s genome is compared with both of his or her parents. This approach raises important questions about responsibilities for communicating parental genome results and determining whether they are relevant to the clinical question at hand. Findings that are incidental, additional, or secondary to the initial clinical question are to be expected and require careful thought and sensitive management.^4^

One of the oldest and most gnarly incidental findings in genetics is that of misattributed parentage, where testing reveals either the father, the mother, or both parents to be genetically unrelated to their child, or not related as stated. The increased use of genome-wide trio sequencing, particularly in pediatrics,^5,6^ will definitively prove biological relationships in a way that previous targeted approaches did not. This can pose a dilemma for the professionals who handle genomic data from trios as to what to do with such information. Some large-scale family-based sequencing studies—such as the Deciphering Developmental Disorders study^7^ and the 100,000 Genomes Project^8^ in the UK—have made explicit statements that they would never reveal information about misattributed parentage. However, the hybrid nature of such studies, which lie at the interface between clinical practice and research, can lead to situations in which such promises clash with views about good clinical practice; e.g., where facts about familial relationships are directly relevant to clinical care. Coupled with the specific aim of such studies to deliver diagnostic results to individual families, routine confirmatory tests may definitively prove something that the clinical teams were not expecting, and they may then feel uncertain about whether, when, and how to communicate these findings.

## MATERIALS AND METHODS

In this paper, we illustrate some of the practical and ethical issues that surround the discovery of misattributed parentage using the following case study:

*Baby Sally has suffered from numerous developmental problems since she was born, and her parents were referred for genetic testing to find out the cause of her disorder before deciding whether to have another child. A molecular diagnosis could improve the management of Sally’s condition, suggest a prognosis, and predict the probability that her parents will have another similarly affected child. Desperate for an answer after numerous investigations have proved uninformative, the family agreed to be enrolled into a research study that would sequence all their DNA and communicate any potential diagnoses. However, the result was ambiguous: a novel variant was found in a gene in which other rare variation is known to cause a severe, dominant developmental disorder similar to Sally’s. Unfortunately, the inheritance of this variant is unknown because the parents’ samples failed analysis. Since this particular variant has not previously been described, it is not certain whether it explains Sally’s signs and symptoms. The interpretation of such a finding is challenging, due to the enormous amount of benign variation across the genome, even within genes that can cause severe disease. However, since both parents are healthy, the variant is only likely to be relevant if it arose spontaneously (a de novo pathogenic variant) and is only present in Sally, rather than inherited from either parent. The clinical team therefore decides to test both parents in their diagnostic laboratory to determine inheritance, and discovers that the variant is not present in either parent but that genetic paternity has been misattributed: Sally’s father is biologically unrelated to her*.

## RESULTS

### Research versus clinical testing

Before we discuss the result of the test in this case and its implications, it is worth pausing to consider two questions that might arise in relation to the decision to perform confirmatory testing.

First, why did the parental samples fail analysis? There are numerous technical reasons why DNA analysis may fail, including sample mixups, low DNA yields, contamination, or poor data quality.^7^ However, familial samples collected for research studies may also fail if they are not genetically related to each other in the expected manner. In a research setting, because such samples generally do not help to answer the overall research question, it is scientifically and economically prudent to exclude unrelated individuals from a family sequencing study as soon as possible to save the cost of full analysis. For this reason, researchers may use an initial genetic screen for quality control to identify sample mixups and unrelated trios (i.e., a genotype-based family analysis),^9^ then declare any unrelated parental samples invalid or failed, but still analyze the child’s genome. This approach also provides a mechanism of information control in studies that have an explicit policy around nondisclosure of misattributed parentage. Because of the different possible reasons a sample may “fail”, this means that the downstream clinical team would not know whether the parental sample was insufficient for analysis or was unrelated to the child unless this was explicitly declared to them.

Second, how should the clinical team act on this research finding? One step undertaken before any research findings are communicated to families is to check they are correct in an accredited diagnostic laboratory. Given that Sally’s clinicians do not know the reason for the parental sample failure, and that a more informative diagnostic result is likely if all three members of the trio are tested, should they now request new samples and proceed to confirm biological parentage (and thus inheritance), as well as the presence or absence of the variant itself? This presents another important question: what information should they provide to the parents about what this testing strategy might reveal? Information about biological parentage would allow the clinical team to interpret the significance of the novel finding in their patient and accurately counsel the parents about their risk of having another affected child. Confirming whether the variant arose de novo or not may be the deciding factor in whether it is considered pathogenic, or indeed diagnostic, in Sally’s case.^10^

A further layer of complexity is added by the fact that these questions arise at the interface between research and clinical care. Any resolution requires careful consideration of the relationship between these two activities, their different motivations, the extent to which they are in practice separable, and the implications for relevant duties of care and lines of responsibility. The question presented by this case and others like it not only concerns what a clinical team should discuss with their patient or patient’s family when supplementary testing to interpret the clinical significance of a finding would also uncover other types of information, but also what researchers should reveal to clinicians and diagnostic laboratories. Knowing when parentage has been misattributed can be extremely useful for deciding which diagnostic testing strategy to use. Do researchers have a responsibility to impart information they hold that might be clinically useful, even if they have declared in their consent materials that they would not reveal such information (in this case, misattributed parentage) to research participants? How should this particular research–clinical boundary be negotiated? Since the discovery of misattributed parentage is unavoidable when performing family-based genetic studies—either through genotype-based quality control procedures or exome/genome sequencing of the trio—a judgment must be made as to how far the information should be propagated along the chain of individuals and institutions involved in the study toward care of the patient.

This example serves to illustrate the difficulty of promising nondisclosure in one setting (research), when in another (clinical care), determining biological parentage can be extremely useful for guiding management. Providing ambiguous information to the clinical team may simply lead them to request new samples and perform their own trio testing, given its potential diagnostic utility. What are they to say at this stage, given that the initial research promised that no information about genetic parentage would be provided?

### Disclosure options

In this case, the finding of misattributed paternity has implications for the certainty of the diagnosis because it means that it is now impossible (with the samples available) to determine whether the single dominant variant is de novo and likely to be pathogenic or has been inherited from the biological father and is likely to be benign. The finding also has implications for any conversation with the parents about the likelihood of this disease occurring in any future child they might have—an issue that is central to cases such as Sally’s, but may not be relevant for everyone. This raises important questions for the clinical team: should they limit their discussion with the family to the likely diagnosis, emphasizing that the chance of this happening in a future child of this couple is extremely low (since they know the variant was not inherited from either of Sally’s stated parents)? Should they also raise the possibility that the true diagnosis has been missed, thus making it impossible to accurately counsel the parents about recurrence risk? Or should they discuss the question of genetic relatedness, and perhaps even attempt to obtain a DNA sample from the ‘genetic father’ to confirm the diagnosis? If they take the former approach, what should they do about recording the information and the test results in Sally’s clinical records, and how should they respond if asked a direct question about genetic paternity (or about reproductive implications) during the consultation? What responsibilities do the clinicians have to be open and honest about all of their findings and interpretations with the parents who have come to them seeking information about Sally’s condition, its cause, and the reproductive implications?

One possibility would be to seek to discuss the result with Sally’s mother alone as she is likely to be aware of the possibility of misattributed paternity. However, were this course of action to be chosen, the information may not be shared with Sally’s father, leaving him to believe, wrongly, that any future pregnancies would potentially be at significant risk when in fact the risk is negligible. This would mean knowingly allowing a patient to leave the clinic with a false belief about his reproductive risk, thereby undermining the autonomy of the man who in the eyes of the law, and indeed the family, is Sally’s father. Furthermore, it would leave the clinicians involved with important information about someone that they had not imparted. Knowing whether or how to document this in the medical records, to ensure it is neither accidentally disclosed nor unnecessarily reinvestigated at some future time, needs careful consideration.

Ethical arguments can be made both for and against disclosure in such cases.^11^ A commonly made argument against disclosure of misattributed parentage is that it is not directly relevant to clinical care. While this is true in many cases, in this case, the clinical relevance to reproductive autonomy is apparent. Another argument made against disclosure is that this information has the potential to undermine the family unit itself, resulting in harmful consequences and potentially leading to violence or abandonment that would not be in the best interests of either the child or the family as a whole. Although such harms are not easily predictable, and harms might also be caused where such information is withheld, this concern is a common response to this type of case. Another argument in favor of nondisclosure is that the family came to the clinic to find the cause of Sally’s condition and understand the risk of having a second affected child, but they did not (or at least not explicitly) come to find out about paternity (for which a test is available directly to the consumer).^12^ Their preferences about receiving this incidental and unsought information, and likely responses to the result, cannot be determined without revealing the concern.

Arguments in favor of disclosure also fall into a number of common positions. It is sometimes argued that not to inform the couple is unjustifiably paternalistic. The couple came to the doctor to seek information about their child’s condition, its causes and its implication for their future reproductive health. The decision not to disclose means that they are not being given the information they seek. Nondisclosure draws on assumptions about the consequences of such disclosure that cannot be foreseen and undermines the couple’s ability to make informed decisions about the future. Failure to disclose may also create confusion where previously there was none, particularly in cases where the couple are already aware of the situation or of its possibility (but have perhaps not realized the relevance to the diagnosis, so not declared it). In this context, withholding the information may simply reduce the chance of making a definitive genetic diagnosis and waste clinical time.

It is clear that many of the arguments both for and against disclosure (or nondisclosure) are grounded in concerns about its impact on children and their families. However, data regarding the relative probability of these harms or benefits are largely absent, suggesting the need for caution in their use in policy-making and practice without further research. In the absence of a convincing evidence base, some have argued that the primary duty is to avoid harm (a duty of nonmaleficence).^13^ This argument seems more convincing in a research context and, indeed, the consensus has generally been that the discovery of misattributed parentage ought not to be disclosed in a research setting where it is not relevant to the research question. Mandava et al. argue that “nondisclosure should be the default position for researchers”.^14^ In clinical practice, the issue has not previously arisen routinely because the use of parental testing to interpret a child’s result was rare until the advent of genomic medicine. Nevertheless, in the clinical setting, in contrast with that of research, recommendations over the past few decades have tended to emphasize the importance of explaining the potential of genetic testing to reveal family structure at the time of consent, and to be open and honest about results that have clinical significance.^11^ There are, however, a range of viewpoints in the literature,^15–17^ and despite an increased focus on patient-centered medicine,^18^ the management of misattributed parentage remains challenging. Some, such as Palmore et al.,^19^ argue for a clause on the consent form “that any incidental information about parentage will not be revealed”. However, they do not consider cases where this policy would directly obfuscate information about risks for future children.

It is our view that clinical significance should determine whether or not to disclose results in the clinic. While there are situations in which nondisclosure is justifiable because it is clear that the finding of misattributed parentage has no clinical implications, it is unlikely to be justified in cases where inheritance patterns are crucial to the interpretation of a result, or for the delivery of clear and honest information about reproductive choice. Where there is a possibility of finding and disclosing misattributed parentage—either through clinical or research uses of genomics—this should be addressed during the consent or pretest counseling process.^20^ The fact that genomic trio testing will determine biological relatedness—and therefore also detect misattributed parentage—should be made explicit through counseling, patient information sheets, and/or consent forms in any situation where it might affect a patient’s clinical management. Although disclosure policies may vary, couples can then make an informed decision as to whether they are content to go ahead with testing.

Since misattributed parentage is relatively rare, a delicate balance must clearly be struck between managing the expectations of families and avoiding causing unnecessary distress to the vast majority of people to whom it will be an irrelevant possibility. Prevalence estimates vary from 1 to 30% (the Nuffield Council of Bioethics cites a “current best estimate of 1–3%” from a range of studies,^21^ while a meta-analysis found a median of 4% across 17 studies),^22^ although those with the highest figures have demonstrable ascertainment bias, such as samples sent for paternity testing. Conversely, parents who know they are genetically unrelated to their child may decide against sending a DNA sample to family sequencing studies if the unavoidable discovery of misattributed parentage is explained upfront. A robust, open, and honest discussion could therefore substantially reduce both the prevalence and severity of any downstream harms.

## DISCUSSION

The issue of misattributed parentage is not new, but our ability to discover it routinely is increasing in both quality and quantity as a result of high-throughput DNA sequencing technologies. This is further highlighted in situations, such as the case of baby Sally above, where the achievement of clarity about inheritance patterns (and hence about parentage) is core to the purpose of testing and determining a diagnosis. It is therefore crucial that genomic researchers and clinicians carefully consider how they will manage this unavoidable finding in the joint territory they increasingly inhabit. Both good research governance and good clinical practice demand consideration of the relative harms and benefits of disclosing information beyond the scope of the original inquiry. Clinical laboratories must carefully weigh the potential harms of testing genetic relatedness directly with the potential benefits of making a definitive diagnosis and facilitating reproductive counseling on a case-by-case basis. Ensuring that patients and families are aware of the possibility of revealing misattributed parentage before consenting for testing is also important for respecting individual autonomy and minimizing downstream harms. The fact that sensitive communication of information about misattributed parentage can be challenging is not a good enough reason to avoid such discussions.

Finally, the issue of misattributed parentage has wider implications for responsible data management. Since most genomic sequencing data are deposited into shared databases to facilitate research,^23^ perhaps the worst possible scenario would be a policy of nondisclosure of misattributed parentage to the clinical team and family, followed by unintentional disclosure and accidental discovery. Since it is an entirely anticipatable incidental finding, studies must therefore have a policy addressing the identification of misattributed parentage and an ethical framework for how the data will subsequently be handled.

In conclusion, for hybrid research studies that routinely use trio sequencing and communicate results to clinicians, difficulties will necessarily arise if there is an explicit nondisclosure policy relating to misattributed parentage. Researchers must take exceptional care not to inadvertently reveal such information, and clinicians must decide whether to adhere strictly to the study’s policy or use their clinical judgment on a case-by-case basis. Where the information has clear clinical utility and relates directly to the purpose of testing (such as reproductive counseling), we recommend this is discussed upfront during the consenting process, and suggest that future policies should allow for more case-specific judgment around disclosure.
